# Effects of acyl-coenzyme A binding protein (ACBP)/diazepam-binding inhibitor (DBI) on body mass index

**DOI:** 10.1038/s41419-021-03864-9

**Published:** 2021-06-09

**Authors:** Adrien Joseph, Hui Chen, Gerasimos Anagnostopoulos, Léa Montégut, Antoine Lafarge, Omar Motiño, Maria Castedo, Maria Chiara Maiuri, Karine Clément, Safae Terrisse, Anne Laure Martin, Ines Vaz-Luis, Fabrice Andre, Franziska Grundler, Françoise Wilhelmi de Toledo, Frank Madeo, Laurence Zitvogel, François Goldwasser, Benoit Blanchet, Frédéric Fumeron, Ronan Roussel, Isabelle Martins, Guido Kroemer

**Affiliations:** 1 Centre de Recherche des Cordeliers, Equipe labellisée par la Ligue contre le cancer, Inserm U1138, Université de Paris, Sorbonne Université, Paris, France; 2 Metabolomics and Cell Biology Platforms, Institut Gustave Roussy, Villejuif, France; 3 Faculté de Médecine, Université de Paris Saclay, Kremlin Bicêtre, Paris, France; 4 INSERM, NutriOmics Research Unit, Sorbonne Université, Paris, France; 5 Assistance Publique Hôpitaux de Paris, Nutrition Departement, Pitié-Salpêtrière Hospital, Sorbonne Université, 47-83 bd de l’Hôpital, 75013 Paris, France; 6 Department of Medical Oncology, Saint-Louis Hospital, Paris Descartes University, AP-HP, Paris, France; 7 Direction des Datas, Unicancer, Paris, France; 8 INSERM Unit 981, Gustave Roussy, Cancer Campus, Villejuif, France; 9 Medical Oncology, Gustave Roussy, Cancer Campus, Villejuif, France; 10 Buchinger Wilhelmi Clinic, Überlingen, Germany; 11 Institute of Molecular Biosciences, NAWI Graz, University of Graz, Graz, Austria; 12 Field of Excellence BioHealth, University of Graz, Graz, Austria; 13 BioTechMed-Graz, Graz, Austria; 14 INSERM U1015, Gustave Roussy, Cancer Campus, 94800 Villejuif, France; 15 INSERM CICBT1428, Centre d’Investigation Clinique—Biothérapie, 94800 Villejuif, France; 16 Department of Medical Oncology, Cochin Hospital, AP-HP, Paris, France; 17 URP4466, Paris University, Paris, France; 18 Pharmacokinetics and Pharmacochemistry Unit, Cochin Hospital, Paris Descartes University, CARPEM, AP-HP, Paris, France; 19 UMR8038 CNRS, U1268 INSERM, Faculty of Pharmacy, University of Paris, PRES Sorbonne Paris Cité, CARPEM, 75006 Paris, France; 20 Centre de Recherche des Cordeliers, UMR-S 1138, INSERM, Université de Paris, Paris, France; 21 Department of Diabetology, Endocrinology, Nutrition, AP-HP, Bichat Hospital, Paris, France; 22 Institut Universitaire de France, Paris, France; 23 Department of Physiology, University Complutense of Madrid, Madrid, Spain; 24 Pôle de Biologie, Hôpital Européen Georges Pompidou, AP-HP, Paris, France; 25 Suzhou Institute for Systems Medicine, Chinese Academy of Medical Sciences, Suzhou, China; 26 Karolinska Institute, Department of Women’s and Children’s Health, Karolinska University Hospital, Stockholm, Sweden

**Keywords:** Obesity, Diagnostic markers

## Abstract

In mice, the plasma concentrations of the appetite-stimulatory and autophagy-inhibitory factor acyl-coenzyme A binding protein (ACBP, also called diazepam-binding inhibitor, DBI) acutely increase in response to starvation, but also do so upon chronic overnutrition leading to obesity. Here, we show that knockout of *Acbp/Dbi* in adipose tissue is sufficient to prevent high-fat diet-induced weight gain in mice. We investigated ACBP/DBI plasma concentrations in several patient cohorts to discover a similar dual pattern of regulation. In relatively healthy subjects, ACBP/DBI concentrations independently correlated with body mass index (BMI) and age. The association between ACBP/DBI and BMI was lost in subjects that underwent major weight gain in the subsequent 3–9 years, as well as in advanced cancer patients. Voluntary fasting, undernutrition in the context of advanced cancer, as well as chemotherapy were associated with an increase in circulating ACBP/DBI levels. Altogether, these results support the conclusion that ACBP/DBI may play an important role in body mass homeostasis as well as in its failure.

## Introduction

Acyl-coenzyme A binding protein (ACBP), also called diazepam-binding inhibitor (DBI) is a phylogenetically ancient protein with two distinct functions^1,2^. As an intracellular protein, ACBP/DBI binds, buffers and transports medium-size acyl-coenzyme A molecules, hence contributing to lipid metabolism^3,4^. As an extracellular protein, ACBP/DBI binds to γ-aminobutyric acid (GABA) receptors of the A type (GABA_A_R)^5,6^, and at least within the central nervous system, to other neurotransmitter receptors (such as the ODN receptors) as well^7,8^. Hence, ACBP/DBI participates in paracrine and neuroendocrine signaling^4^.

ACBP/DBI is a leaderless protein, meaning that it undergoes nonconventional secretion (bypassing the Golgi apparatus) and rather depends on autophagy, as this has been initially found in fungi^9,10^ and then confirmed in human and mouse cells^11,12^. The protein is ubiquitously expressed by nucleated cells (https://www.proteinatlas.org). In yeast (*Saccharomyces cerevisiae*), it is required for sporulation^13^. In nematodes (*Caenorhabditis elegans*), it favors pharyngeal pumping^13^, correlating with the fact that, in fruit flies (*Drosophila melanogaster*), one of the ACBP/DBI orthologues stimulates mouth hook movement, which is the equivalent of mastication^14^. Intriguingly, knockout of ACBP/DBI orthologues in yeast or nematodes increases lifespan^15,16^, suggesting that the protein may have pro-aging effects, a conjecture that has not yet been investigated in vertebrates. Of note, in mice and human cells, ACBP/DBI inhibits autophagy^12^, which is a well-studied antiaging mechanism^17–19^.

In mice, intravenous or intraperitoneal injection of recombinant ACBP/DBI protein or its transgenic overexpression in the liver stimulates eating behavior through GABA_A_R signaling^20^. Moreover, three distinct manipulations designed to reduce ACBP/DBI levels, namely (i) an inducible whole-body knockout of the *Acbp/Dbi* gene, (ii) injection of neutralizing antibodies against ACBP/DBI, or (iii) induction of autoantibodies through an autovaccination strategy, similarly rendered mice resistant against weight gain induced by high-fat diet^12^. ACBP/DBI neutralization also prevented high-fat diet-induced glucose intolerance and hepatosteatosis. Importantly, anti-ACBP/DBI antibodies blunted food intake after a 24 h fasting period. In addition, extensive biochemical and metabolomic analyses suggest that ACBP/DBI is not only a potent appetite stimulator but also favors lipo-anabolic reactions^12^.

In humans, plasma concentrations of ACBP/DBI correlate with body mass index (BMI)^12,20,21^. Thus, ACBP/DBI levels are abnormally low in patients with anorexia nervosa^12,21^, but high in patients with obesity^12,20^. Thus, ACBP/DBI is among the rare appetite-stimulatory factors that increases in human obesity^20,22^, contrasting with many other appetite-stimulatory mediators (as prominently exemplified by ghrelin) that are rather reduced in non-syndromic obesity^23,24^. Hence, ACBP/DBI could be causally involved in human obesity, a hypothesis that requires further investigation.

The regulation of plasma ACBP/DBI levels in mice follows complex rules in which short-term and long-term effects must be distinguished. Shortly after starvation (no access to food, water at libidum), circulating ACBP/DBI concentrations increase due to the autophagy-dependent release of pre-synthesized intracellular ACBP/DBI into the extracellular space. Since ACBP/DBI is an inhibitor of autophagy and an inducer of appetite, it acts as the executor of a “hunger reflex”^25^. Of note, chronic overnutrition of mice with a high-fat diet (which suffices to stimulate obesity) or with a normal diet (as this occurs in leptin-deficient *Ob/Ob* mice, which are hyperphagic) causes an increase in ACBP/DBI expression at the mRNA and protein levels in major metabolic organs including the liver and white adipose tissue, correlating with increased ACBP/DBI plasma levels^12^.

Based on these premises, we decided to investigate ACBP/DBI levels in several different cohorts of patients that were either on long-term trajectories of weight gain or weight loss, as well as patients that underwent acute perturbations by voluntary fasting, advanced cancer diagnosis and chemotherapy. Altogether, our results confirm that the rules governing ACBP/DBI concentrations in human are similar to those observed in mice.

## Results

### ACBP/DBI inhibition abolishes high-fat diet-induced weight gain in mice

**Fig. 1:**
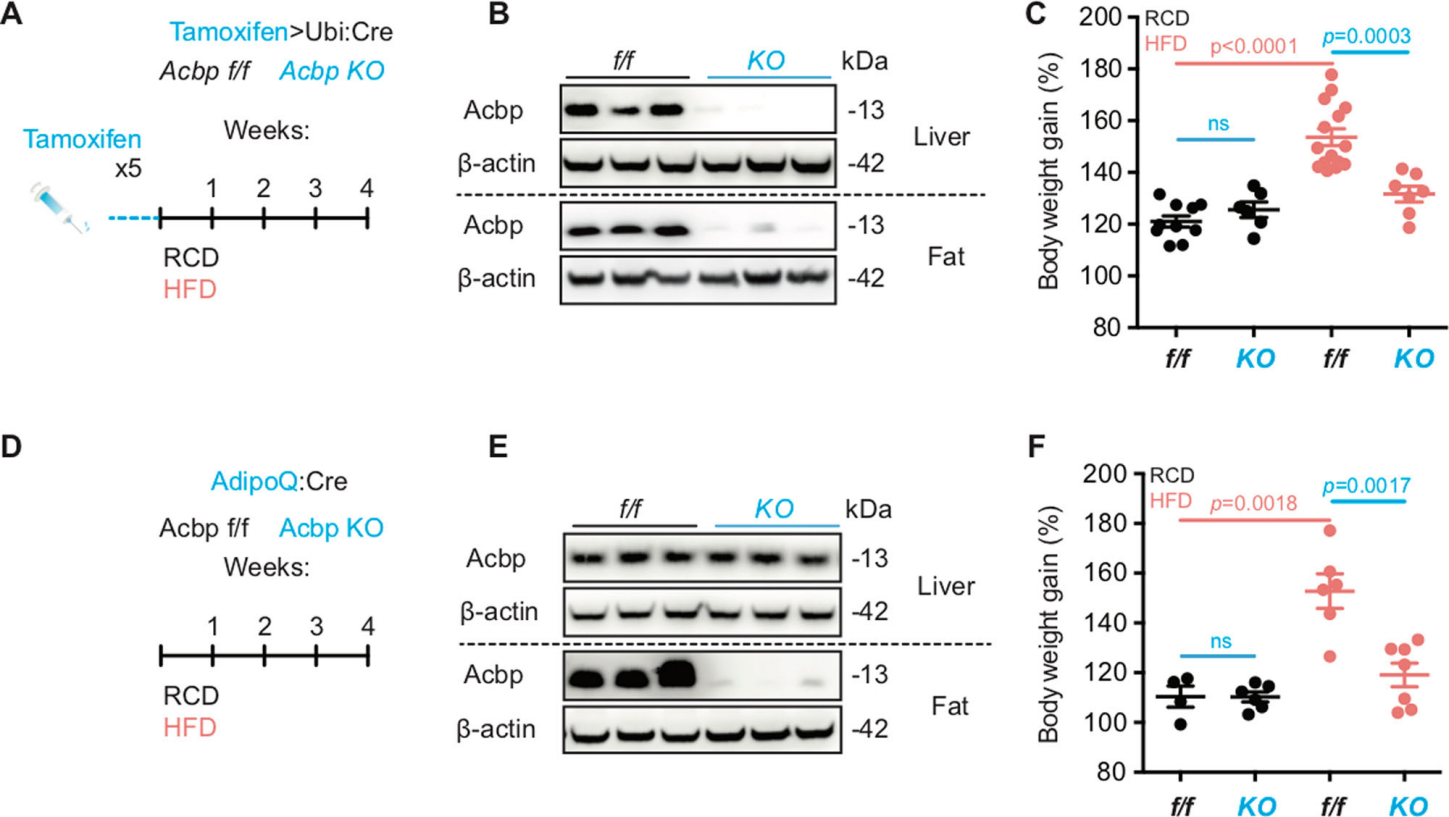
Genetic ablation of ACBP confers resistance against high-fat diet-induced weight gain. **A** Schematic representation of whole-body inducible ACBP knockout murine model in regular chow diet (RCD) or high-fat diet (HFD) regime for 4 weeks (*ubi* = ubiquitin, *Acbp*^f/f^ = *Acbp*^flox/flox^ control mice, *Acbp* KO = *Acbp*^flox/flox^; *ubiCre* mice having received five daily tamoxifen injections to activate Cre and to excise the floxed *Acbp* exon 2). Both controls and *Acbp*^flox/flox^; *ubiCre* similarly received tamoxifen injections. **B** Representative immunoblots of ACBP and β-actin from liver and white adipose-tissue (fat) protein lysates in ACBP whole-body knockout (KO) and control (f/f) littermates. **C** Body weight of ACBP whole-body knockout (KO) and control (f/f) littermates receiving either RCD or HFD regime for 4 weeks. Statistical comparison between the two types of regimes (RCD, HFD) as well as between the two types of genetic backgrounds (f/f, KO) was performed via two-tailed unpaired Student’s *t* test (ns nonsignificant). Each point represents one mouse. **D** Schematic representation of adipocyte-specific ACBP knockout murine model in RCD or HFD regime for 4 weeks (*Acbp*^f/f^ = *Acbp*^flox/flox^ control mice, *Acbp* KO = *Acbp*^flox/flox^; A*dipoQCr*e-positive mice). **E** Representative immunoblots of ACBP and β-actin from liver and white adipose-tissue (fat) protein lysates in ACBP adipocyte-specific knockout (KO) and wild-type (f/f) littermates. **F** Weight measurement in ACBP adipocyte-specific knockout (KO) and control (f/f) littermates receiving either RCD or HFD regime for 4 weeks. Statistical comparison was performed by means of the two-tailed unpaired Student’s *t* test (ns nonsignificant).

In adult mice, ACBP/DBI can be genetically removed by flanking exon 2 of the *Dbi* gene with flox sites and excising this exon by a tamoxifen-inducible Cre recombinase^12^. Systemic injection of tamoxifen causes ubiquitous expression of Cre with the subsequent knockout of *Dbi* throughout the body (Fig. 1A). Mice lacking ACBP/DBI following this manipulation in all organs (exemplified for liver and white adipose tissue, Fig. 1B) become resistant to weigh gain induced by high-fat diet (Fig. 1C). A similar weigh gain-resistant phenotype was observed for mice in which ACBP/DBI was constitutively removed from adipocytes only (Fig. 1D–F). Of note, neither the inducible whole-body *Acbp/Dbi* knockout nor the adipocyte-specific constitutive knockout of *Acbp/Dbi* resulted in a skin phenotype (Fig. S2). We did not detect any histological change between the skin of adipocyte-specific *Acbp/Dbi* knockout (*AdipoQ:Acbp KO*) and control (*AdipoQ:Acbp f/f*) mice (Fig. S3). Thus, at difference with a recent report dealing with the constitutive knockout of ACBP in all cells or the conditional knockout of ACBP in keratinocytes^26^, ACBP/DBI inhibition caused resistance against the metabolic consequences of high-fat diet without compromising the epidermal barrier.

### Correlations between ACBP/DBI plasma levels and body mass index in a large cohort

**Fig. 2:**
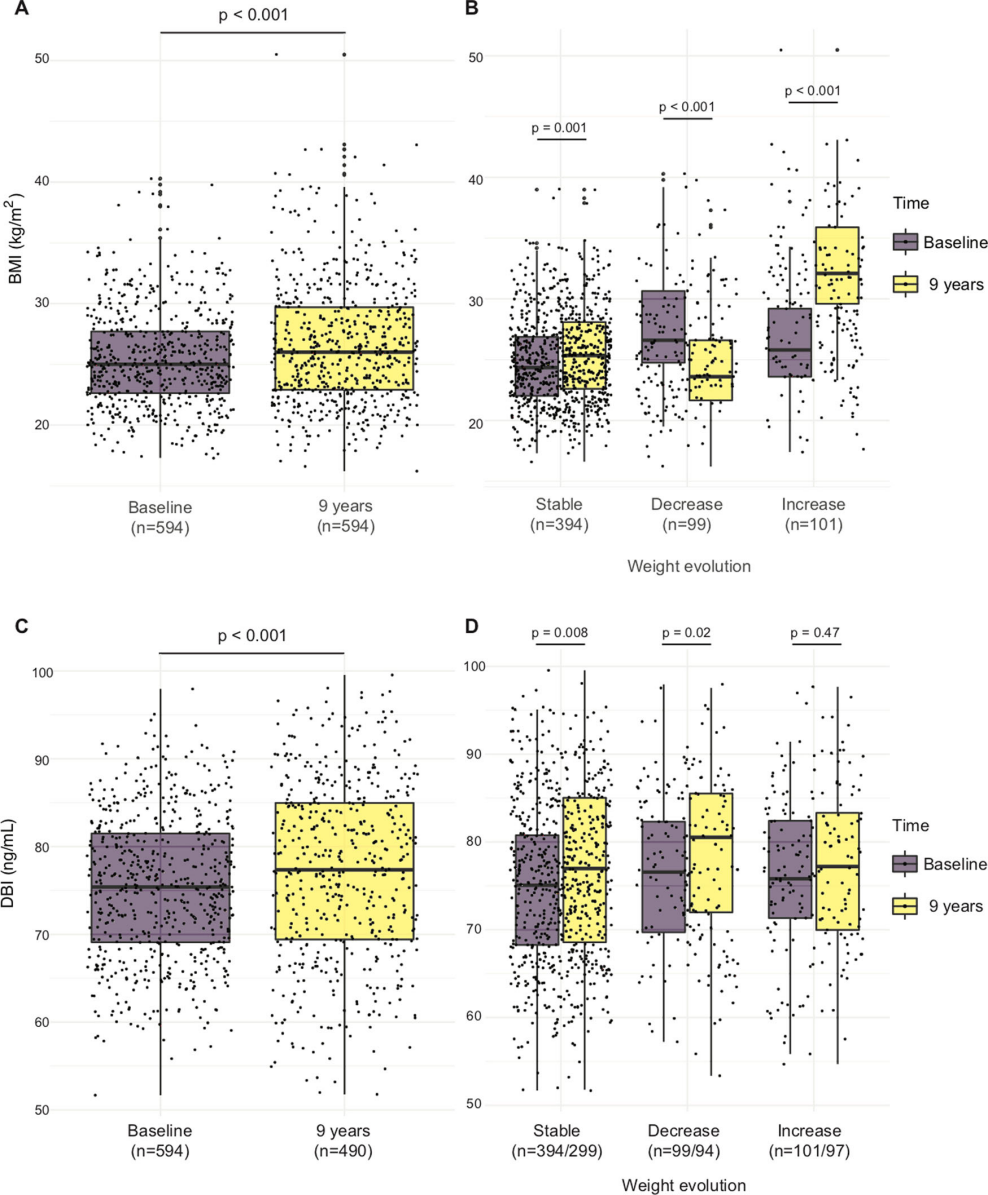
Evolution of BMI and ACBP/DBI levels in individuals with decreasing, stable or increasing body weight. The evolution of BMI (**A**, **B**) and ACBP/DBI (**C**, **D**) is shown for individuals who gain weight, lose weight or whose weight remains stable over a 9-year period. Box and whisker plots representing BMI (kg/m^2^) (**A**, **B**) and ACBP/DBI levels (ng/mL) (**C**, **D**) at baseline and 9 years later in the DESIR cohort.

**Fig. 3:**
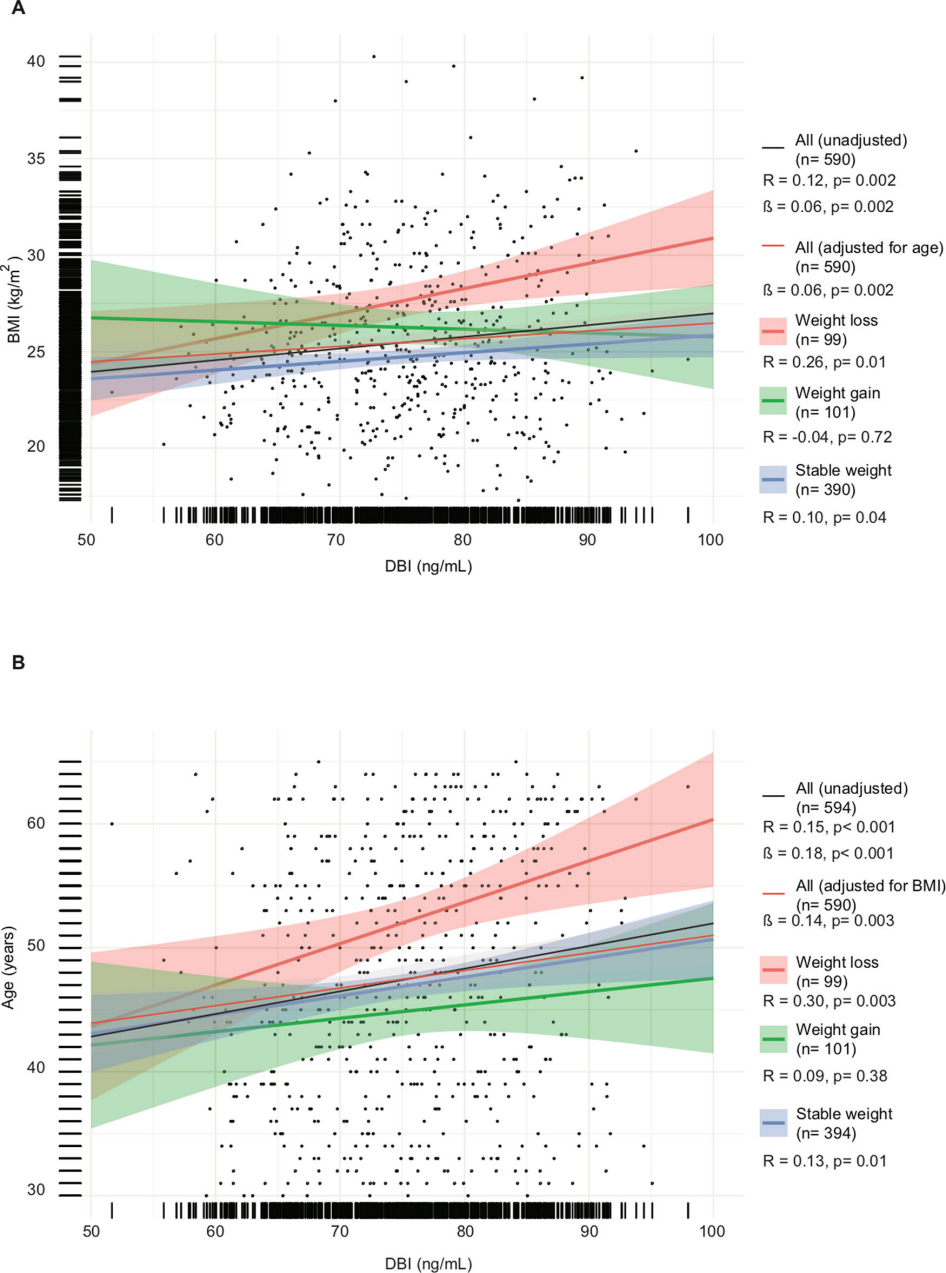
ACBP/DBI correlates independently with BMI and age except in patients with subsequent weight gain. Scatter plot with regression line between ACBP/DBI (ng/mL) and body mass index (kg/m^2^) (**A**) or age (**B**) in patients who lose weight (≥5%), gain weight (≥7%), or remain stable (variations <2%) in the DESIR cohort. Pearson’s correlation coefficient (R) and their *p* value, number of samples available (*n*) and estimates (ß) from a linear model before and after adjustment are shown in the legend of each panel.

We took advantage of the DESIR cohort that recruited 5212 outpatients from the Loire valley between 1994 and 2010 with longitudinal follow-up every 3 years to determine variations in diabetes-relevant and cardiometabolic parameters. We included 99 patients who—over a 9-year period—lost weight (by >5%), 101 patients who gained weight (by >7%) and 394 control patients without major weight variations [between −2% and +2%] (Fig. 2A, B). The characteristics of the patients from the different cohorts are described in Table S1. In these samples, ACBP/DBI levels were quantified by means of a commercial ELISA (Fig. 2C, D). We found a significant (*p* < 0.001, Pearson) correlation between plasma ACBP/DBI concentrations and BMI over the entire group of patients (*N* = 590), and this correlation was independent from age (*p* = 0.04) (Fig. 3A). A meta-analysis of several independent cohorts with available clinical data showed that ACBP/DBI significantly correlated with systolic blood pressure (Fig. S4A) and triglyceride levels (Fig. S4B) but tended to be negatively associated with renal function (Fig. S4C). The association between these variables and ACBP remained significant after adjustment for BMI for triglyceride levels (adjusted OR = 2.90 [1.48–5.69], *p* = 0.002) and estimated glomerular filtration rate (eGFR, adjusted OR = 0.97 [0.94–0.99], *p* = 0.006) but less so for systolic blood pressure (adjusted OR = 1.03 [1.00–1.06], *p* = 0.07).

Of note, for patients who maintained a stable weight or lost weight, the correlation between ACBP/DBI levels and BMI was significant. However, for those individuals who gained weight, the correlation between plasma ACBP/DBI and initial BMI was lost (Fig. 3A). A similar observation was made for the correlations between ACBP/DBI and age: a significant correlation for all groups, independently from BMI, a good correlation for the subgroups with stable or decreasing weight, but a loss of correlation for the subgroup with weight gain (Fig. 3B).

Altogether, these findings indicate that physiological alterations that precede a major weight gain affect the correlation between ACBP/DBI and BMI or age. For comparison, we measured the plasma levels of α-Klotho, which has been suggested to act as a major antiaging factor^27,28^. We did not find any significant correlation between α-Klotho levels and chronological age (Fig. S5A), BMI (Fig. S5B) or ACBP/DBI levels (Fig. S5C).

### Perturbation of the correlation between ACBP/DBI and body mass index by fasting and cancer

**Fig. 4:**
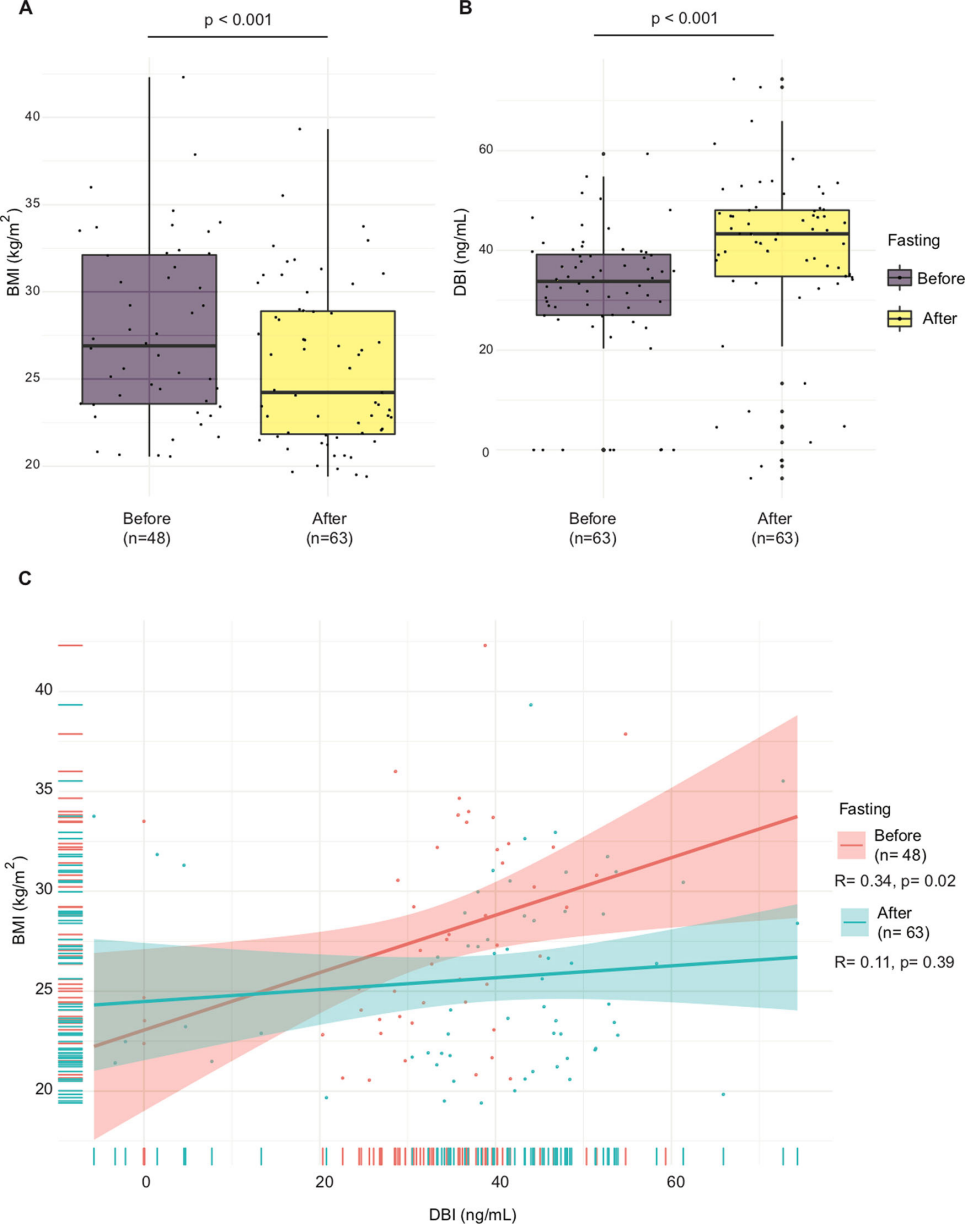
Effects of fasting on ACBP/DBI levels. Fasting induces a decrease in BMI (**A**), an increase of ACBP/DBI (**B**) and a loss of the correlation between ACBP/DBI and BMI (**C**). Box and whisker plots representing BMI (kg/m^2^) (**A**) and ACBP/DBI levels (ng/mL) (**B**) before and after fasting in the BWC cohort. Scatter plots with regression lines between ACBP/DBI (ng/mL) and body mass index (kg/m^2^) (**C**) before and after fasting in the BWC cohort. Pearson’s correlation coefficient (R) and their *p* value and number of samples available (*n*) are shown in the legend of each panel. Note that the results of paired *t* tests limited to patients with complete BMI data (before and after fasting, *n* = 48) show a similar difference in terms of BMI decrease (*p* < 0.001) and DBI increase (*p* < 0.001).

In the next step, we measured ACBP/DBI plasma concentrations in individuals who volunteered to undergo prolonged fasting (4–16 days) as an approach to improve cardiometabolic features and well-being^29^. Of note, fasting reduced BMI (Fig. 4A) but induced an increase in ACBP/DBI levels (Fig. 4B). Moreover, ACBP/DBI plasma levels and BMI correlated at baseline, and this correlation was lost upon fasting (Fig. 4C).

**Fig. 5:**
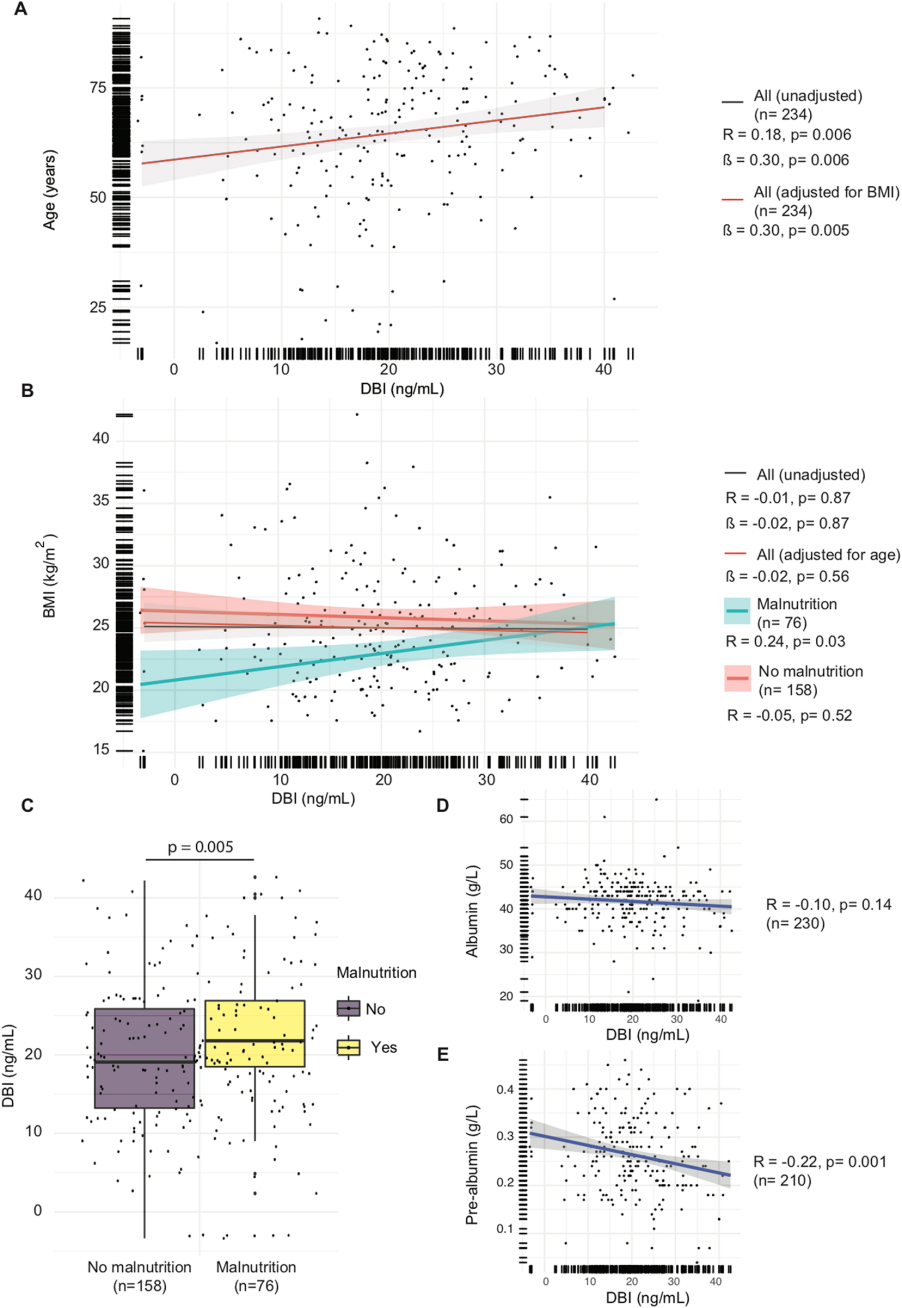
ACBP/DBI correlates with age but not with BMI in patients with advanced cancer, excepted for undernourished patients. Scatter plots with regression lines between ACBP/DBI (ng/mL) and age (**A**) and BMI (**B**) before and after adjustment for BMI (**A**) and age (**B**) and in patients with and without malnutrition (**B**), and between ACBP/DBI (ng/mL) and albumin (**D**) and prealbumin (**E**) in the advanced cancer cohort. Pearson’s correlation coefficient (R) and their *p* value, number of samples available (*n*) and estimates (ß) from a linear model before and after adjustment are shown in the panels. Box and whisker plots represent ACBP/DBI levels (ng/mL) in patients with and without malnutrition (**C**).

In addition, in a cohort of patients with advanced cancer, no correlation was found between ACBP/DBI levels and BMI, although ACBP/DBI levels did correlate with age (Fig. 5A, B). Importantly, those patients with undernutrition (defined as BMI < 18.5 kg/m^2^ or albumin levels <35 g/L) did exhibit a correlation between ACBP/DBI and BMI and actually exhibited higher ACBP/DBI concentrations than non-undernourished patients (Fig. 5C). For the whole population of cancer patients, we observed an inverse correlation of ACBP/DBI levels and prealbumin (but not albumin) levels (Fig. 5D, E), knowing that prealbumin levels are particularly sensitive to an acute reduction in protein ingestion and overall caloric intake^30^.

In conclusion, it appears that voluntary fasting and acute disease-associated undernourishment can cause an elevation of ACBP/DBI.

### Relationship between ACBP/DBI levels and chemotherapy

**Fig. 6:**
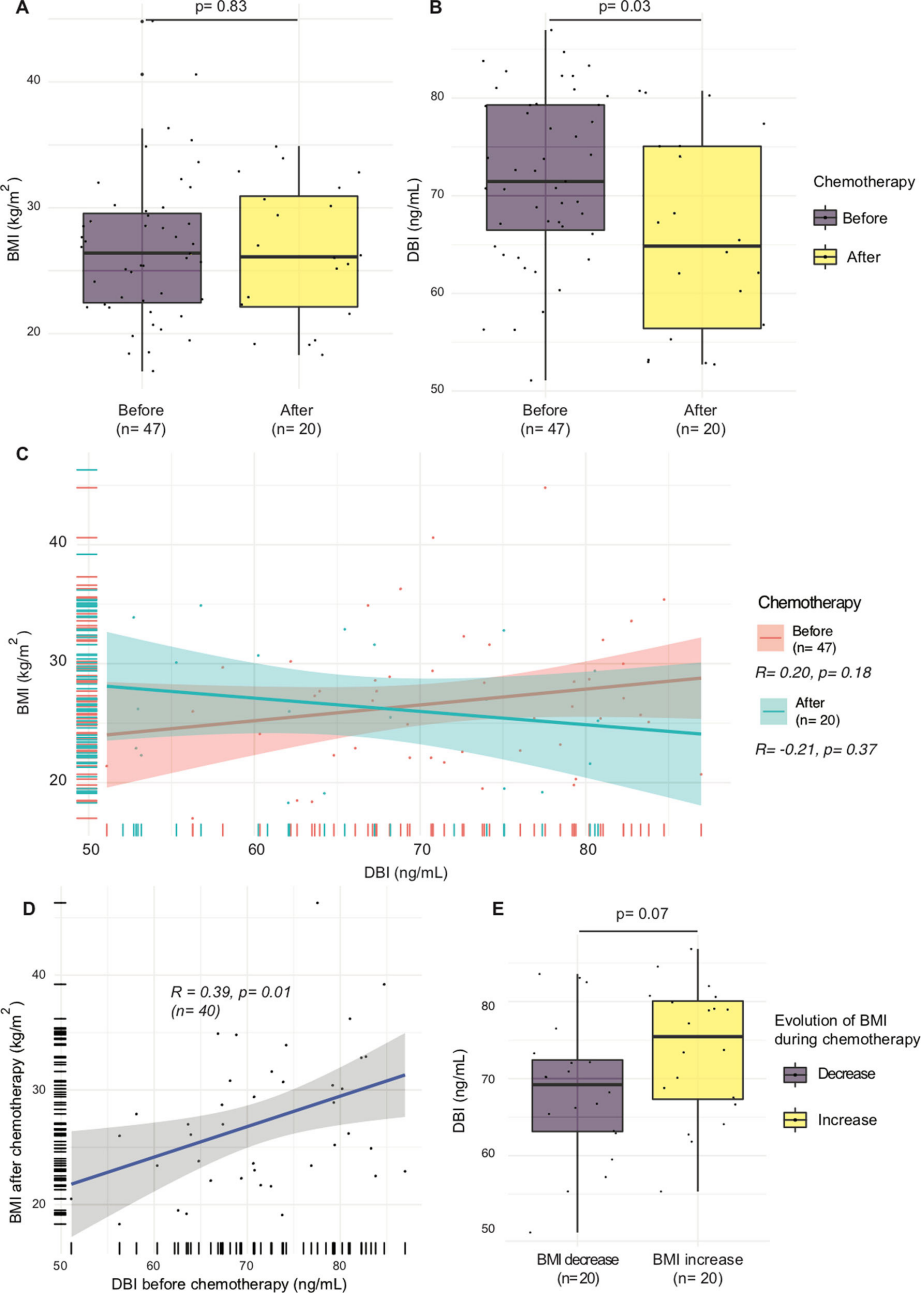
Effects of chemotherapy on ACBP/DBI levels in breast cancer patients. Chemotherapy does not induce any change in BMI (**A**), but induces a decrease of ACBP/DBI (**B**), as well as a loss of the correlation between ACBP/DBI and BMI (**C**). ACBP/DBI better correlates with BMI after chemotherapy compared to before chemotherapy (**D**) and patients whose weight increased during chemotherapy tend to have higher DBI levels (**E**). Box and whisker plots representing BMI (kg/m^2^) (**A**) and ACBP/DBI levels (ng/mL) (**B**) before and after chemotherapy in the CANTO cohort. Scatter plots with regression lines between ACBP/DBI (ng/mL) and body mass index (kg/m^2^) are shown before and after chemotherapy for the CANTO cohort (**C**). Pearson’s correlation coefficient (R) and their *p* value and number of samples available (*n*) are shown in the legend of each panel.

We found an absence of ACBP/DBI-BMI correlation for 37 samples from the CANTO study (Cancer toxicities, NCT01993498), a French prospective cohort of patients with nonmetastatic breast cancer (stage I–III) designed to quantify and generate predictors of chronic toxicities related to treatment^31^. Of note, after chemotherapy, the ACBP/DBI levels fell (Fig. 6A, B). Although no significant correlation between ACBP/DBI and BMI was found before and after chemotherapy in this small cohort (Fig. 6C), the levels of ACBP/DBI before chemotherapy significantly correlated with BMI after chemotherapy (Fig. 6D). Baseline DBI tended to be higher in patients who gained weight after chemotherapy (Fig. 6E) and was significantly associated with weight gain after adjustment for initial BMI (*p* = 0.04).

**Fig. 7:**
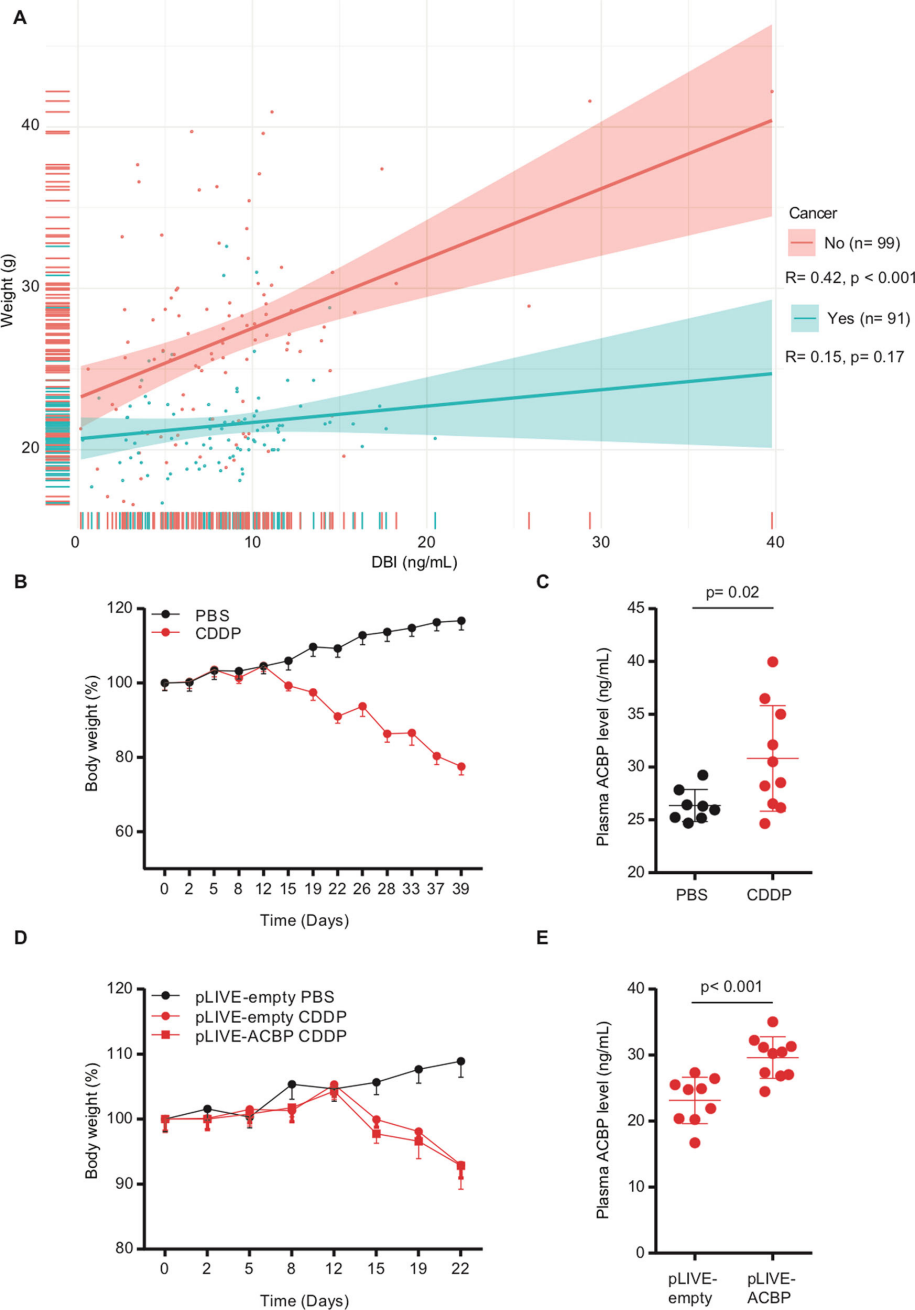
Anorexigenic effects of chemotherapy in mice. **A** Scatter plot with regression line between ACBP/DBI (ng/mL) and weight (g) in mice with and without cancer. Pearson’s correlation coefficient (R) and their *p* value and number of samples available (*n*) are shown in the legend of each panel. **B**, **C** Metabolic effects in anorexic mice due to chemotherapy. Mice were treated with PBS or cisplatin (CDDP: 4 mg/kg) every week for 40 days. Body weight was monitored (**B**) and plasma ACBP levels were quantified on day 39 (**C**) (*n* = 8–10 mice per group). Body weight PBS versus CDDP *p* < 0.001. **D**, **E** Metabolic effects of ACBP expression in anorexic mice due to chemotherapy. Mice received hydrodynamic injections of empty pLIVE or pLIVE-ACBP (100 µg) expressing vector at day 2. Three days after, mice were treated with PBS or cisplatin (CDDP: 4 mg/kg) once per week. Body weight was monitored (**D**) and plasma ACBP concentrations were measured in the CDDP treated mice at day 8 (**E**) (*n* = 8–10 mice per group). Body weight pLIVE-empty PBS versus pLIVE-empty CDDP *p* < 0.001. Body weight pLIVE-empty PBS versus pLIVE-ACBP CDDP *p* = 0.75. Body weight pLIVE-empty CDDP versus pLIVE-ACBP CDDP *p* < 0.001.

In mice, we observed a positive ACBP/DBI-BMI correlation for cancer-free individuals but not for tumor bearers (Fig. 7A). Chemotherapy of tumor-free mice with cisplatin led to anorexia (Fig. 7B), as well as an increase in ACBP/DBI plasma concentrations (Fig. 7C). Hydrodynamic injection of a vector that causes the liver-specific expression of mouse *Acbp/dbi* led to an increase in circulating ACBP/DBI levels (Fig. S6A) as well as a reduction in plasma glucose levels in otherwise untreated mice (Fig. S6B), demonstrating that ACBP/DBI was bioactive. However, the elevation of circulating ACBP/DBI did not reverse the chemotherapy-induced weight loss (Fig. 7D, E).

Altogether, these results indicate that chemotherapy as such has a potent effect on body composition as well as on ACBP/DBI levels. Artificial elevation of ACBP/DBI to supranormal levels is not sufficient to improve chemotherapy-induced anorexia.

## Discussion

Prior studies in mice suggest that the levels of circulating ACBP/DBI are affected by two different regulatory circuitries^12^. First, starvation for 1–2 days causes an autophagy-dependent release of intracellular ACBP/DBI into the extracellular space, resulting in an increase in ACBP/DBI levels, allowing ACBP/DBI to act as a peripheral stimulator of food intake. This represents a homeostatic feedback loop acting in the short term. As shown here, the acute reduction in body weight induced by chemotherapy also results in an elevation of ACBP/DBI plasma levels. Second, weight gain induced by high-fat diet or overconsumption of a normal diet (as this occurs in leptin-deficient *Ob*/*Ob* mice) results in an upregulation of ACBP/DBI mRNA and protein expression in the liver and white adipose tissue, correlating with elevated circulating ACBP/DBI concentration as well. It appears plausible that this obesity-associated elevation in ACBP/DBI contributes to maintaining a constant phase of overeating, thus perpetuating the obese state in a pathogenic feed forward loop^12,25^. Thus, in mice, two apparently opposed conditions, starvation and obesity are able to stimulate a similar increase in the appetite-stimulatory factor ACBP/DBI.

The data presented here, support the contention that, in humans, ACBP/DBI is regulated following a similar dual pattern. Voluntary fasting or disease-associated undernutrition (as this occurs in advanced cancer and upon chemotherapy) results in an augmentation of ACBP/DBI plasma concentrations. In contrast, in close-to-healthy steady-state conditions, ACBP/DBI levels correlate positively with BMI. This correlation is lost after fasting, chemotherapy, as well as in the context of advanced cancer, as illustrated by a meta-analysis of all cohorts in which we measured ACBP/DBI plasma concentrations (Fig. S7A). Similar trends are observed for the correlation between ACBP/DBI and age across different cohorts (Fig. S7B). In this context, it appears intriguing that the positive correlation between DBI and BMI or age was lost among those patients that subsequently underwent a major weight gain. Whether this is related to erratic eating behaviors among persons at risk of weight gain and/or reflects a functional disruption of the homeostatic circuitry involving ACBP/DBI remains an open question that deserves further investigation.

Intriguingly, ACBP/DBI independently correlates with both chronological age and BMI, knowing that obesity is (one of) the most important age-accelerating factor(s)^32,33^. It will be interesting to measure the correlation between DBI and biological (rather than chronological) aging, as determined by laboratory parameters (such as telomere length and the epigenetic clock) or clinical indicators (such as the imminent manifestation of major age-related diseases). Hence, future studies should determine whether ACBP/DBI correlates with biological age better than it does with chronological age. Indeed, the literature on non-human model organisms cited in the Introduction indicates that ACBP/DBI is a pro-aging molecule. In this context, it appears intriguing that ACBP/DBI plasma levels are elevated in Alzheimer’s disease^34^. Whether it will be possible to inhibit ACBP/DBI with the scope of obtaining similar antiaging effects in mammals as in yeast and nematodes, however, remains a matter of speculation.

Beyond the human data that are central to this paper, our experimental results on mice support the contention that eliminating ACBP/DBI from white adipose tissue is sufficient to confer protection against high-fat diet-induced weight gain. Future studies will have to determine whether removal of ACBP/DBI from other metabolic organs (such as the liver or skeleton muscle) will have similar effects, or whether these effects must be ascribed to cell-autonomous ACBP/DBI effects on pre-adipocyte differentiation^35^. It has been reported that constitutive ACBP/DBI knockout affecting the whole animal or the epithelial layer of the skin alone affects the barrier function of the epidermis and that this would account for obesity resistance^26^. At odds with this postulate, however, inducible ACBP/DBI knockout in adulthood or adipocyte-specific knockout did not provoke any skin phenotype, yet protected against weight gain induced by high-fat diet. These findings echo prior observations showing that autoimmunization against ACBP/DBI (to induce autoantibodies capable of neutralizing extracellular ACBP/DBI) or repeated injection of monoclonal antibodies specific for ACBP/DBI can reduce food intake and weight gain, again without any manifest skin phenotype^12^. These results underscore the notion that ACBP/DBI neutralization can be safely achieved to obtain favorable metabolic effects.

In summary, the results presented here support the existence of two opposed regulatory systems determining ACBP/DBI concentrations in the plasma. Short-term alterations resulting from voluntary fasting or a disease-related caloric deficit elevate ACBP/DBI levels. In contrast, in steady-state conditions, ACBP/DBI levels increase with BMI and age, a correlation that is lost in multiple pathological conditions including future weight gain, morbid obesity, advanced cancer, or chemotherapy. Importantly, in ambulatory outpatients without major health issues, ACBP/DBI plasma concentrations positively correlate with triglyceride levels and systolic blood pressure. Whether measures to reduce ACBP/DBI including neutralizing antibodies may ameliorate such parameters indicative of metabolic syndrome constitutes an open question for future preclinical and clinical research.

## Materials and methods

### Plasma ACBI/DBI and α–Klotho measurements in human cohorts

Plasma ACBP/DBI levels were measured using the KA0532 ACBP (Human) ELISA kit in seven different cohorts.

The DESIR (*Données Épidémiologiques sur le Syndrome d’Insulino-Résistance*) study^36^ is a 9-year prospective cohort including 5212 volunteers from the general population at ten health examination centers in western France. Biological samples were taken and BMI were measured every 3 years.

For ACBI/DBI measurements, we selected individuals who gained (+7%, *n* = 101) or lost weight (−5%, *n* = 99) during their 9 years follow-up and compared each of them to 2 control individuals whose weight remained stable [between −2% and +2%, *n* = 394]. Plasma ACBP/DBI was measured at baseline and at 9 years in these patients.

The BWC (Buchinger Wilhelmi Clinic) study^29^ is an observational study in which 1422 patients were included in a fasting program. This program was supervised at the BWC (Überlingen, Germany) and began after a physical examination and exclusion of predefined contraindications. Patients were given a 600 kcal vegetarian diet on the day before the fasting period and then an average of 200–250 kcal/d for a minimum of 4 days (fasting period). For ACBI/DBI measurements, 63 randomly selected patients had their plasma ACBP/DBI measured before and at the end of the fasting program.

The advanced cancer cohort^37^ included unselected consecutive patients (*n* = 280) with various solid tumors admitted from December 2014 to November 2015 to an outpatient chemotherapy unit at Cochin Hospital (Paris, France).

The CANTO trial (for CANcer TOxicities NCT01993498)^31^ is a prospective clinical cohort funded by the French research agency (ANR-10-COHO-0004-CANTO) including nonmetastatic early-stage breast cancer (*n* = 12,000), with the primary objective to assess chronic toxicity related to treatment (surgery, chemotherapy, radiation therapy, hormone therapy, etc…). CANTO is coordinated by UNICANCER, the National Cooperative Group of French Cancer Centers. The study was approved by the national regulatory authorities and ethics committee (ID-RCB: 2011-A01095–36, 11–039). All patients enrolled in the sub-study CANTO-Oncobiome (76 patients) provided written informed consent, including consent for the biological data collection.

Patients whose plasma was collected before (*n* = 47) and after (*n* = 20) neoadjuvant/adjuvant chemotherapy and whose BMI was measured were included in our study.

The sampling method from these four cohorts is pictured in Fig. S1.

The Anorexia and Obese cohorts have been published previously^12^. Briefly, they include patients suffering from anorexia nervosa (*n* = 57) and age- and sex-matched controls (*n* = 14) and obese patients before and 12 months after bariatric surgery (*n* = 110 + 14 controls in a first cohort and *n* = 39 in a second cohort).

Plasma α-Klotho levels were measured using the human SAKL (Soluble alpha-Klotho) ELISA Kit (# MBS7606772–96) in the DESIR cohort.

### Conditional whole-body ACBP/DBI knockout mice

Whole-body ACBP/DBI knockout mice were generated as previously described^12^. In brief, Acbp fl/fl mice (Bentley, WA, USA) were crossed with B6.Cg-Tg (UBC-cre/ERT2)1Ejb/1J mice (Jackson Laboratory, Bar Harbor, ME, USA). Cre recombinase was activated with tamoxifen (75 mg/kg of body weight i.p./mouse on a daily basis for 5 days). Prior to administration, tamoxifen was diluted in corn oil (90%) and ethanol (10%) up to the final concentration of 20 mg/ml.

### Ubiquitous adipose-tissue ACBP/DBI knockout mice

Adipocyte-specific ACBP/DBI knockout mice were generated by crossing Acbp fl/fl mice (loxP sites flanking Acbp exon 2; Bentley, WA, USA) with B6;FVB-Tg(AdipoQ-cre)1Evdr/J mice (Jackson Laboratory).

### Skin histology

Mouse skin tissue samples were harvested from *Acbp/Dbi*^*f*/f^; AdipoQ *Cre*^+/−^ mice followed by fixation in 20 mL 4% v/v paraformaldehyde solution (4 °C). After 24 h, the samples were dehydrated by incubation in gradually increasing ethanol solutions (70–100% v/v), followed by paraffin inclusion. Five μm sections were stained using hematoxylin and eosin and scanned by means of a Zeiss Lame Axioscan (objective: ×20). Images were analyzed using the Zen software.

### Mouse experiments

Mice were bred and housed in a pathogen-free, temperature-controlled environment with 12 h light/dark cycles according to the FELASA guidelines, EU Directive 63/2010 and French legislation. Wild-type C57BL/6 mice were purchased from Envigo (Envigo, Gannat, France). *Sat1-KO* mice are a gift from Prof. Leena Alhonen, University of Eastern Finland^38^.

To test the association between cancer-bearing and cancer-free mice, we isolated plasma from males (*n* = 92) and females (*n* = 98) mice from different age (9–48 weeks old) and genotypes: wild-type (*n* = 154), *AdipoQ:Acbp KO* (*n* = 16), *Fpr1-KO* (*n* = 14), and *Sat1-KO* (*n* = 6). Tumors were made from different cell lines, namely Lewis Lung cancer (*n* = 20), MCA205 fibrosarcoma (*n* = 60), R80 cholangiocarcinoma (*n* = 6), TC-1 non-small cell lung cancer cells (*n* = 10), injected subcutaneously (Lewis Lung cancer, MCA205 and R80), or orthotopically (TC-1). Mice were fed with normal (*n* = 180) or high-fat diet (*n* = 50) with water ad libitum.

For the chemotherapy-induced anorexia model, 6-week-old female wild-type C57BL/6 received water and food ad libitum. Mice were treated with PBS or cisplatin (CDDP: 4 mg/kg once per week) for 40 days by i.p. administration. Mouse plasma was harvested from blood collection tubes by centrifugation at 15,000 rpm for 30 min, and ACBP levels were determined by ELISA (MBS2025156; from MyBioSource, San Diego, CA, USA) as instructed by the manufacturer.

To study the effect of ACBP inhibition on high-fat diet-induced weight gain, 8–10-week-old male C57BL/6 mice received normal or high-fat chow diet (Safe #260 HF) and water ad libitum. Weight was monitored on a weekly basis over the course of 4 weeks.

Prior to picture capturing, mice were individually housed and acclimatized for 5 min in the 25 × 15 × 10 cm cage. Mice of all genotypes were individually photographed in the same graduated cage.

To induce in vivo ACBP/DBI expression, mice were injected intravenously with pLIVE cDNA (100 µg) in saline (10% of the body weight) via the tail vein. DNA injection was completed in less than 5 s.

All animal experiments were conducted in compliance with the local Animal Experimental Ethics Committee (protocols #27952, #19607, #24938, #18967, #4022, #24410, #25355, and #24410).

### Immunoblotting

20–25 µg of protein lysates (either from liver or from epididymal white adipose tissue) were separated by SDS-PAGE (4–12% Bis-Tris acrylamide gels, WG1402BOX, Thermo Fisher), and electro-transferred to immunoblot PVDF membranes (1620177, Biorad) according to standard procedures. Membranes were blocked (5% w/v nonfat powdered milk) for 2 h followed by overnight incubation in a primary antibody mixture (murine ACBP, ab231910, Abcam). After overnight incubation, membranes were washed, followed by 1 h incubation in horseradish peroxidase (HRP)-labeled secondary antibody mix (4050-05, Southern Biotech). Membranes were washed and revealed in the ImageQuant™ LAS 4000 (GE Healthcare). β-actin primary antibody (ab49900, Abcam) was used as loading control.

### Statistical analysis

Continuous variables are described by mean and standard deviation and categorical variables as number and percentage. ACBP/DBI measurements across groups or in sequential samples are represented as box and whisker plots (mean, first and third quartiles) and the relation between DBI and other continuous parameters are represented as dots with one or more regression lines according to subgroups.

ACBP/DBI concentrations were compared with unpaired Student’s *t* test across groups and with paired *t* tests for sequential measurements in the same patients. Pearson’s correlation coefficients with their *p* values were calculated. To illustrate the association between two continuous variables after adjustment for a third, we give the estimates (ß) from a linear regression models before and after adjustment and plot the adjusted regression line with predictor against fitted values from this model. The number of samples available is featured on each plot. Weight curves were longitudinally analyzed with type II ANOVA and pairwise comparisons using the Tum-Growth software tool (https://kroemerlab.shinyapps.io/TumGrowth/).

To have an estimate of the correlation coefficient across different cohorts, we did a meta-analysis of all cohorts we published, using Fisher’s *z* transformation of correlations. Pearson’s correlation coefficients from each study and the pooled estimate are represented with their 95% confidence interval in forest plots. Heterogeneity was assessed using the *I*^2^ statistic. Because of the expected heterogeneity of the cohorts pooled, including patients from various settings (oncology, bariatric surgery, anorexia, fasting, and healthy) all meta-analyses were performed using a conservative random-effects model. Except for BMI, meta-analyses were performed across cohorts for which the variables of interest were available.

Each test was two-sided and a *p* value below 0.05 was considered significant. Statistics were managed using R software version 3.6.0 (R Foundation for Statistical Computing, Vienna, Austria; https://www.R-project.org/).

## Supplementary information

Supplemental files

Figure S1

Figure S2

Figure S3

Figure S4

Figure S5

Figure S6

Figure S7
