# Supplementary material for: Effects of acyl-coenzyme A binding protein (ACBP)/diazepam-binding inhibitor (DBI) on body mass index
Source: Cell Death Dis. 2021 Jun 9;12(6):599. doi: 10.1038/s41419-021-03864-9 (PMC8190068; doi:10.1038/s41419-021-03864-9)
Supplement: Supplementary file 1 — Supplemental files [file 41419_2021_3864_MOESM1_ESM.docx]

|  | **BWC** | **DESIR** | | | | **Advanced cancer** | **CANTO** |
| --- | --- | --- | --- | --- | --- | --- | --- |
|  |  | All | Weight loss | Weight gain | Stable weight |  |  |
| n | 63 | 594 | 99 | 101 | 394 | 280 | 47 |
| Age (mean (SD)) | 60.3 (9.4) | 47.5 (10.1) | 52.4 (9.5) | 45.0 (9.7) | 46.9 (10.0) | 64.9 (14.8) | 51.7 (8.4) |
| Female sex (%) | 40 (63.5) | 326 (54.8) | 57 (57.6) | 72 (71.3) | 197 (49.9) | 107 (38.2) | 47 (100) |
| Baseline BMI (kg/m2) (mean (SD)) | 27.7 (5.2) | 25.5 (4.0) | 27.8 (4.3) | 26.2 (4.3) | 24.7 (3.6) | 24.9 (4.8) | 26.8 (5.8) |
| BMI after fasting (BWC), at 9 years (DESIR) and after chemotherapy (CANTO) (kg/m2) (mean (SD)) | 25.7 (4.6) | 26.6 (5.1) | 24.4 (4.1) | 32.9 (4.9) | 25.6 (4.0) | - | 27.2 (6.1) |
| Baseline DBI (ng/mL) (mean (SD)) | 31.3 (14.1) | 75.4 (8.3) | 76.3 (8.5) | 76.4 (7.9) | 74.9 (8.3) | 20.16 (9.5) | 71.9 (8.6) |
| DBI after fasting (BWC), at 9 years (DESIR) and after chemotherapy (CANTO) (ng/mL) (mean (SD)) | 39.4 (17.1) | 77.1 (10.3) | 78.5 (9.7) | 77.1 (10.2) | 76.7 (10.6) | - | 65.1 (10.2) |
|  |  | | | | | **Oncological characteristics** | |
| Primary site cancer (%) |  |  |  |  |  |  |  |
| Lung |  |  |  |  |  | 54 (19.3) | 0 (0) |
| Colon |  |  |  |  |  | 21 (7.5) | 0 (0) |
| Prostate |  |  |  |  |  | 30 (10.7) | 0 (0) |
| Breast |  |  |  |  |  | 11 (3.9) | 47 (100) |
| Sarcoma (soft tissue) |  |  |  |  |  | 26 (9.3) | 0 (0) |
| Sarcoma (bone) |  |  |  |  |  | 18 (6.4) | 0 (0) |
| Urinary tract |  |  |  |  |  | 30 (10.7) | 0 (0) |
| Ovary |  |  |  |  |  | 18 (6.4) | 0 (0) |
| Kidney |  |  |  |  |  | 16 (5.7) | 0 (0) |
| Pancreas |  |  |  |  |  | 14 (5.0) | 0 (0) |
| Endometrium |  |  |  |  |  | 7 (2.5) | 0 (0) |
| Liver and biliary tract |  |  |  |  |  | 12 (4.3) | 0 (0) |
| Oesogastric |  |  |  |  |  | 6 (2.1) | 0 (0) |
| Other |  |  |  |  |  | 17 (6.1) | 0 (0) |
| Metastatic disease (%) |  |  |  |  |  | 196 (70.0) | 0 (0) |
| Number of previous therapeutic lines (mean (SD)) |  |  |  |  |  | 0.40 (0.87) | - |
| Albumin (g/L) (mean (SD)) |  |  |  |  |  | 41.31 (5.40) | - |
| Pre-albumin (g/L) (mean (SD)) |  |  |  |  |  | 0.26 (0.08) | - |
| ECOG Scale of Performance Status (%) |  |  |  |  |  |  | - |
| 0-1 |  |  |  |  |  | 197 (70.3) | - |
| 2 |  |  |  |  |  | 76 (27.1) | - |
| 3-4 |  |  |  |  |  | 7 (2.5) | - |
| Malnutrition (%) |  |  |  |  |  | 98 (35.0) | - |
| Adjuvant chemotherapy (%) |  |  |  |  |  | - | 37 (78.7) |
| Union for International Cancer Control stade (%) |  |  |  |  |  | - |  |
| STADE 0 |  |  |  |  |  | - | 5 (10.6) |
| STADE I |  |  |  |  |  | - | 11 (23.4) |
| STADE II |  |  |  |  |  | - | 23 (48.9) |
| STADE III |  |  |  |  |  | - | 6 (12.8) |
| NA |  |  |  |  |  | - | 2 (4.3) |
| **Supplemental table I:** Characteristics of the patients included in the different cohorts  *BWC: Buchinger Wilhelmi Clinic, DESIR: Données Épidémiologiques sur le Syndrome d’Insulino-Résistance, CANTO: Cancer Toxicities, SD: Standard deviation, BMI: Body Mass Index, DBI: Diazepam Binding Inhibitor, ECOG: Eastern Cooperative Oncology Group.* | | | | | | | |

**Supplementary Information**

**Figure S1. Flow diagram representing the sampling from the 4 cohorts.**

**Figure S2. Representative images of adipocyte-specific ACBP knockout (up), and whole-body inducible Acbp knockout (down) murine models receiving regular chow diet (RCD) or high-fat diet (HFD)** (bar scale: 1 cm).

**Figure S3.** **Mouse skin histology.**

Hematoxylin & eosin staining of the skin from adipocyte-specific Acbp knock-out mice (*AdipoQ:Acbp KO*) compared to their control (*AdipoQ:Acbp f/f*) littermates fed with regular-chow (RCD) or high-fat diet (HFD) (Bar scale: 200μM).

**Figure S4. Forest plot representing the correlation between DBI and systolic blood pressure (A), triglyceride (B) and glomerular filtration rate (C) across di­fferent cohorts in which these variables were available.**

*Each Pearson’s correlation coefficient is represented with its 95% confidence interval. The size of the square is proportional to the sample size of the study. The pooled correlation was calculated using a random effect model.*

*Glomerular filtration rate was estimated using the Modified Diet in Renal Disease (MDRD) equation.*

*BWC: Buchinger Wilhelmi Clinic, DESIR: Données Épidémiologiques sur le Syndrome d’Insulino-Résistance.*

**Figure S5. α-Klotho does not correlate with age, BMI or ACBP/DBI.**

Scatter plot with regression line between α-Klotho (ng/mL) and age (A), body mass index (kg/m^2^) (B) or ACBP/DBI (ng/mL) (C) in patients who lose weight (≥5%), gain weight (≥7%), or remain stable (variations < 2%) in the DESIR cohort. Pearson’s correlation coefficient (R) and their p value, number of samples available (n) and estimates (ß) from a linear model before and after adjustment are shown in the legend of each panel.

**Figure S6. Plasma ACBP (A) or glucose (B) levels, after the hydrodynamic injection of PLIVE-empty or PLIVE-ACBP expressing vector (100µg), were monitored at day 8 (n=7 to 8 mice per group).**

**Figure S7. Forest plot representing the correlation between DBI and BMI (A) and age (B) across di­fferent cohorts.**

*Each Pearson’s correlation coefficient is represented with its 95% confidence interval. The size of the square is proportional to the sample size of the study. The pooled correlation was calculated using a random effect model.*

*BWC: Buchinger Wilhelmi Clinic, DESIR: Données Épidémiologiques sur le Syndrome d’Insulino-Résistance, CANTO: Cancer Toxicities*.
