# Supplementary figures and images for: Effects of acyl-coenzyme A binding protein (ACBP)/diazepam-binding inhibitor (DBI) on body mass index
Source: Cell Death Dis. 2021 Jun 9;12(6):599. doi: 10.1038/s41419-021-03864-9 (PMC8190068; doi:10.1038/s41419-021-03864-9)

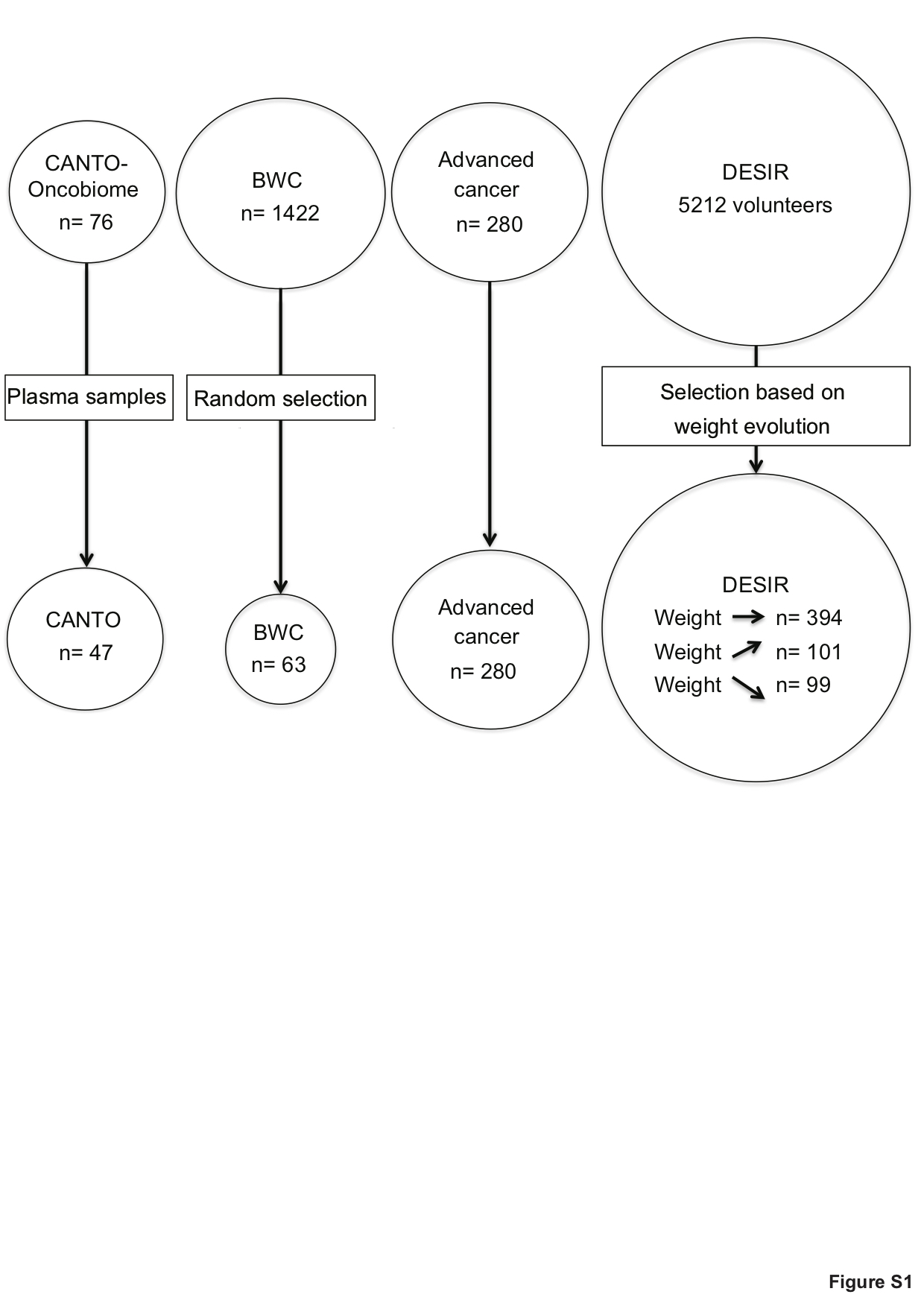

Supplement: Supplementary file 2 — Figure S1 [file 41419_2021_3864_MOESM2_ESM.jpg]

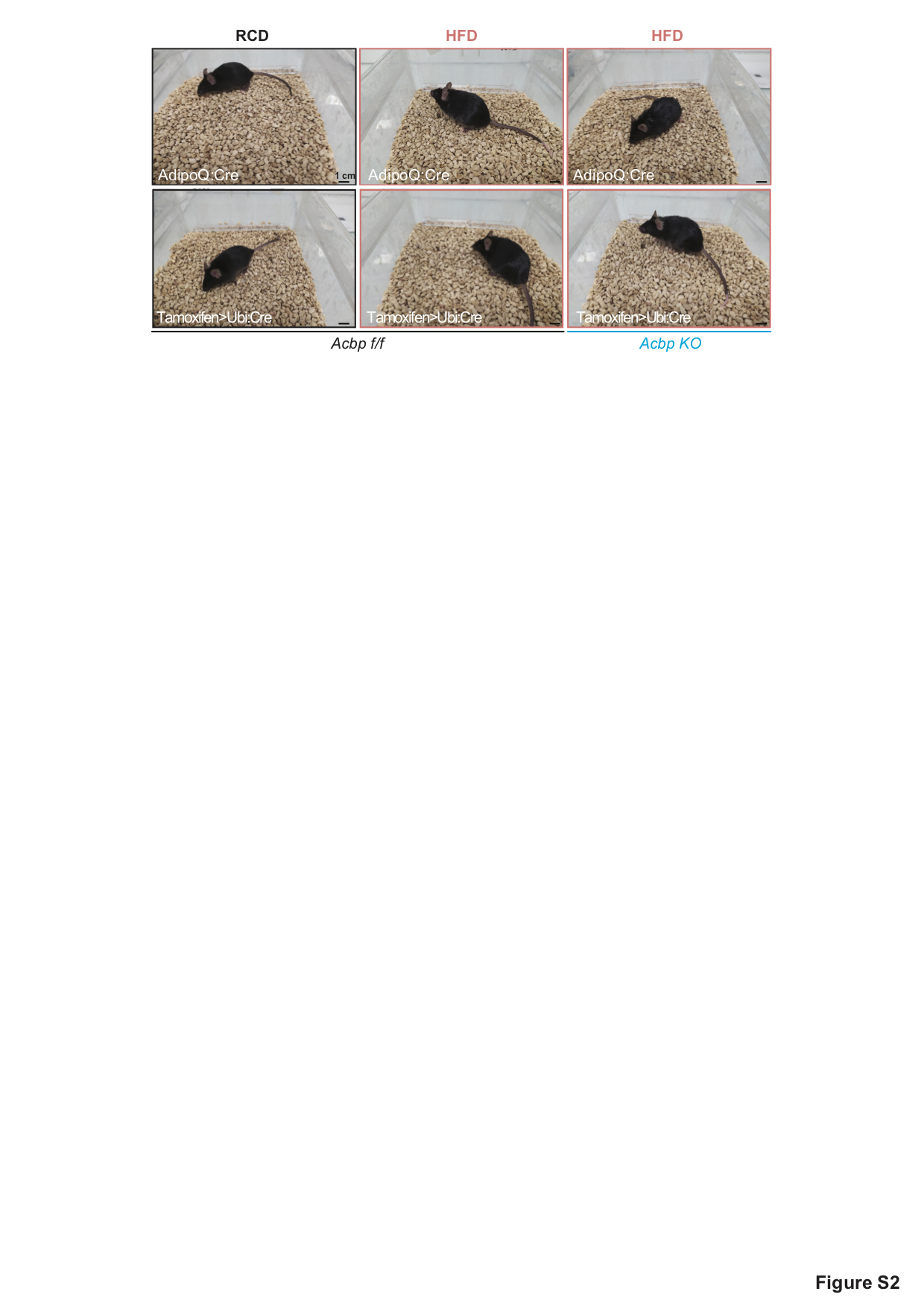

Supplement: Supplementary file 3 — Figure S2 [file 41419_2021_3864_MOESM3_ESM.jpg]

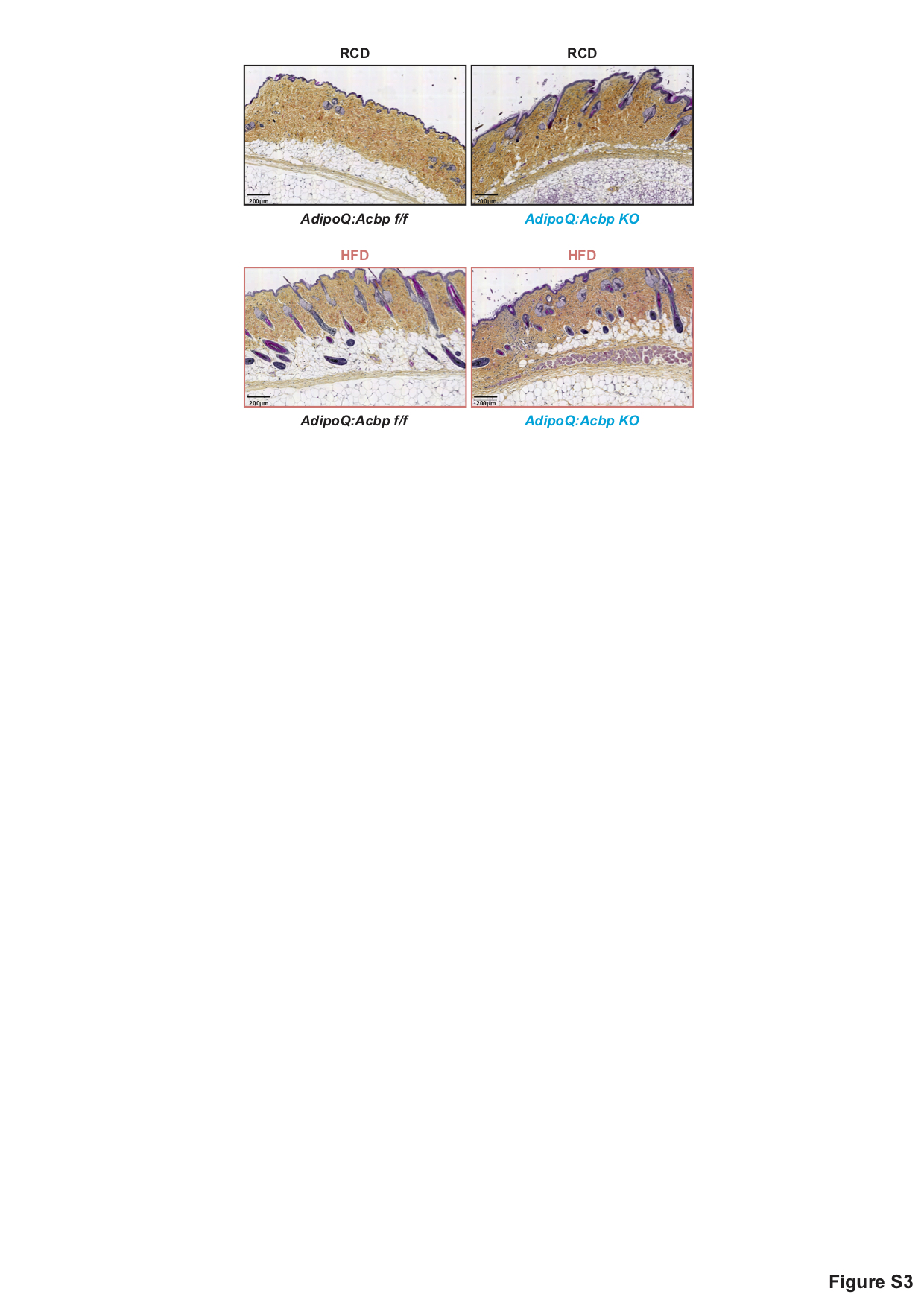

Supplement: Supplementary file 4 — Figure S3 [file 41419_2021_3864_MOESM4_ESM.jpg]

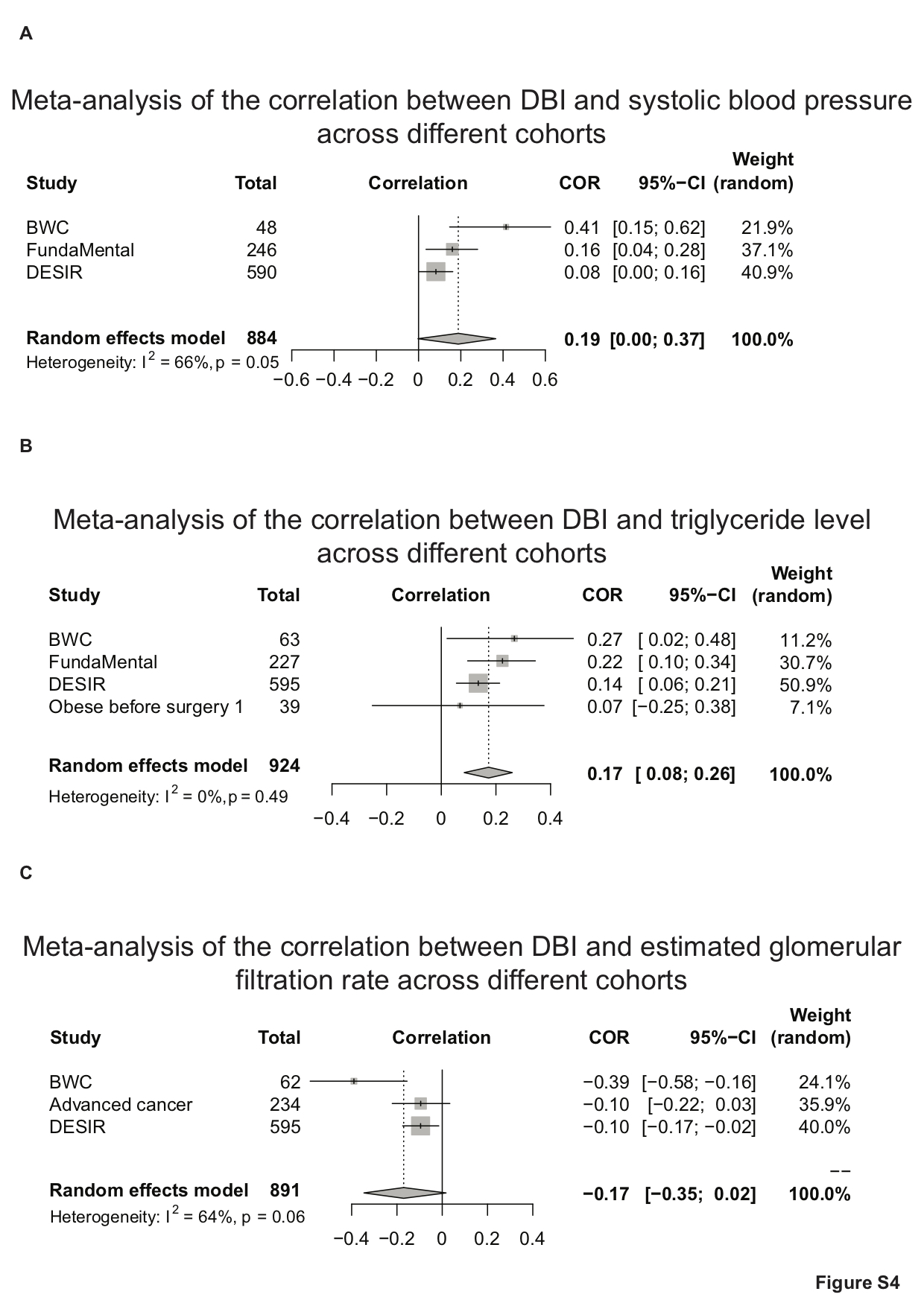

Supplement: Supplementary file 5 — Figure S4 [file 41419_2021_3864_MOESM5_ESM.jpg]

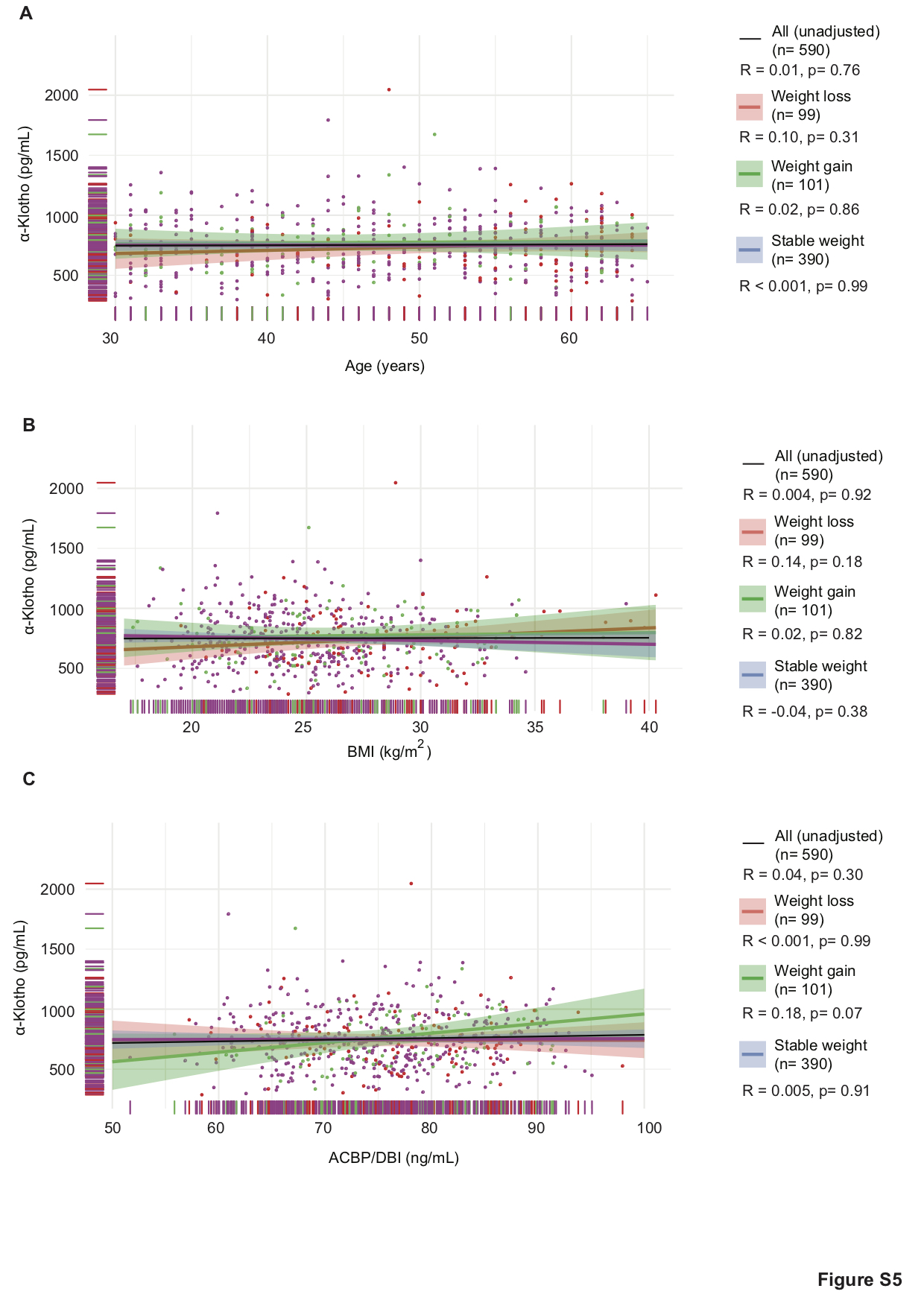

Supplement: Supplementary file 6 — Figure S5 [file 41419_2021_3864_MOESM6_ESM.jpg]

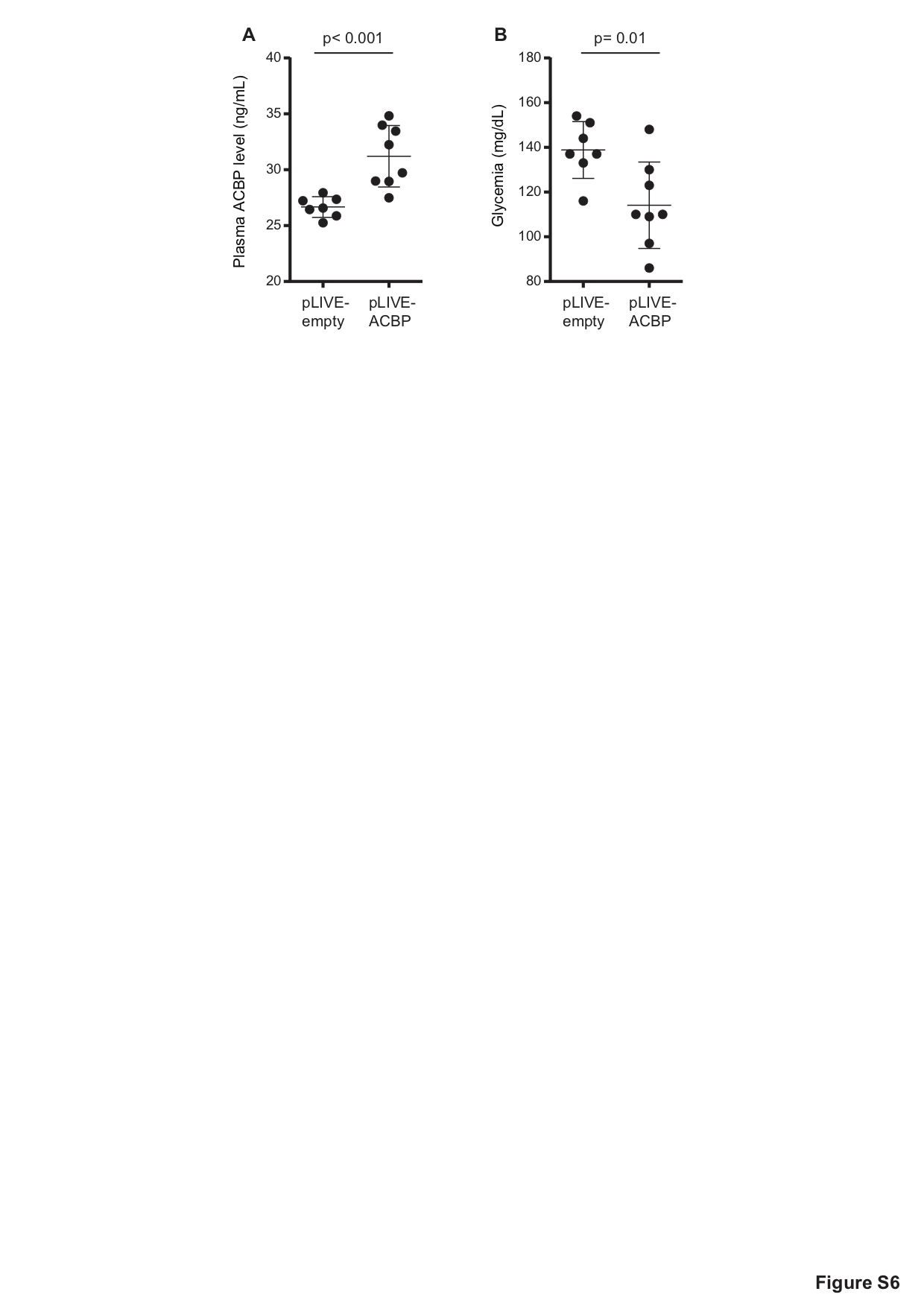

Supplement: Supplementary file 7 — Figure S6 [file 41419_2021_3864_MOESM7_ESM.jpg]

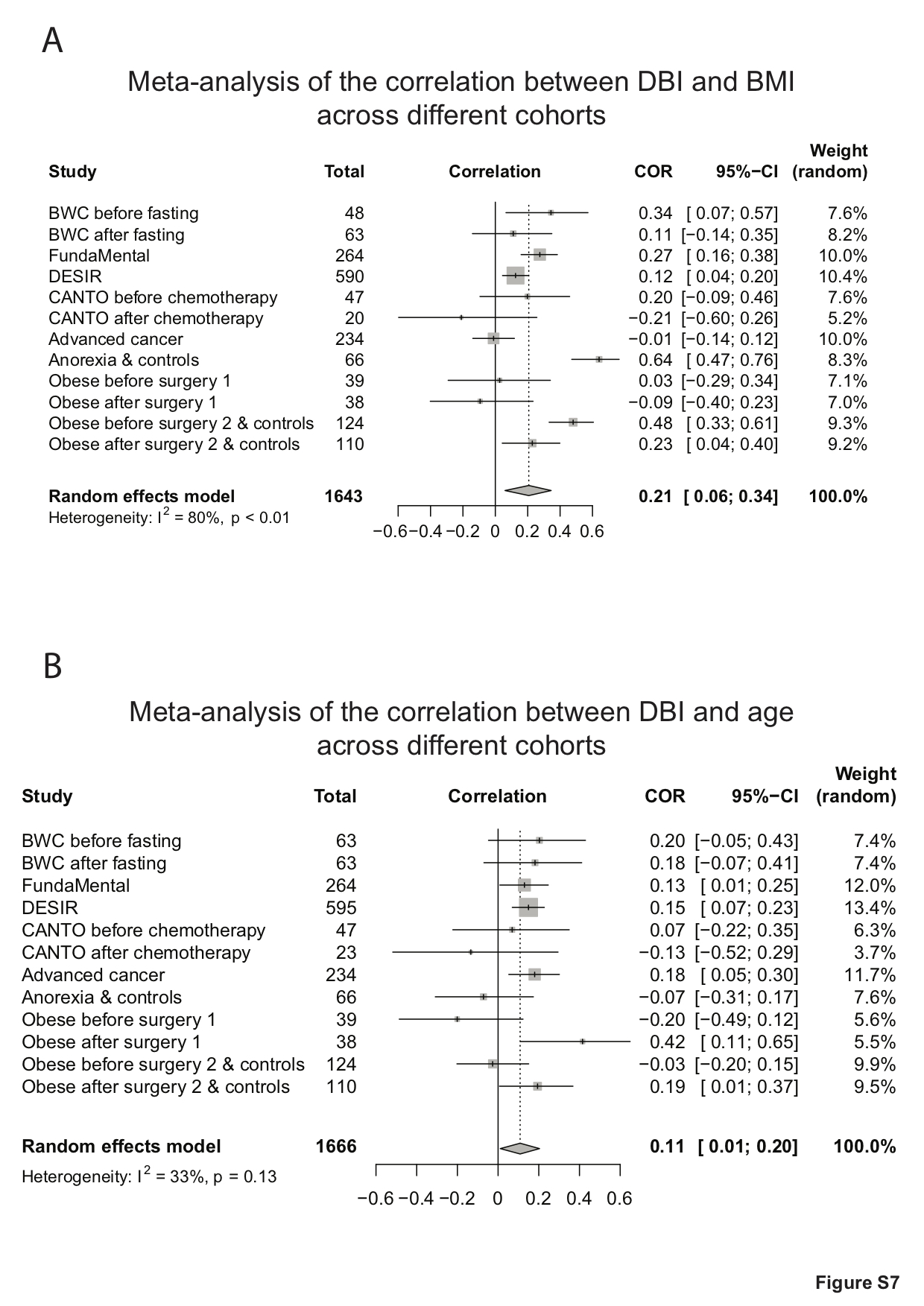

Supplement: Supplementary file 8 — Figure S7 [file 41419_2021_3864_MOESM8_ESM.jpg]
